# 2,4-Bis(3-methoxy­phen­yl)-3-aza­bicyclo­[3.3.1]nonan-9-one

**DOI:** 10.1107/S1600536809050697

**Published:** 2009-12-04

**Authors:** P. Parthiban, V. Ramkumar, Yeon Tae Jeong

**Affiliations:** a Division of Image Science and Information Engineering, Pukyong National University, Busan 608 739, Republic of Korea; b Department of Chemistry, IIT Madras, Chennai, TamilNadu, India

## Abstract

In the crystal structure, the title compound, C_22_H_25_NO_3_, exists in a twin-chair conformation with equatorial orientations of the *meta*-methoxy­phenyl groups on both sides of the secondary amino group. The title compound is a positional isomer of 2,4-bis­(2-methoxy­phen­yl)-3-aza­bicyclo­[3.3.1]nonan-9-one and 2,4-bis­(4-methoxy­phen­yl)-3-aza­bicyclo­[3.3.1]nonan-9-one, which both also exhibit twin-chair conformations with equatorial dispositions of the anisyl rings on both sides of the secondary amino group. In the title compound, the *meta*-methoxy­phenyl rings are orientated at an angle of 25.02 (3)° with respect to each other, whereas in the *ortho* and *para* isomers, the anisyl rings are orientated at dihedral angles of 33.86 (3) and 37.43 (4)°, respectively. The crystal packing is dominated by van der Waals inter­actions and by an inter­molecular N—H⋯O hydrogen bond, whereas in the *ortho* isomer, an inter­molecular N—H⋯π inter­action (H⋯*Cg* = 2.75 Å) is found.

## Related literature

For the synthesis and biological activity of 3-aza­bicyclo­[3.3.1]nonan-9-ones, see: Jeyaraman & Avila (1981 ▶). For the nicotinic acetyl­choline receptor antogonistic activity of diter­penoid/norditerpenoid alkaloids, see: Hardick *et al.* (1996 ▶); Barker *et al.* (2005 ▶). For the structures of the *ortho* and *para* OMe-substitued isomers, see: Parthiban *et al.* (2009*a* ▶); Cox *et al.* (1985 ▶). For related structures, see: Parthiban *et al.* (2008*a* ▶,*b* ▶,*c* ▶, 2009*b* ▶,*c* ▶), Smith-Verdier *et al.* (1983 ▶); Padegimas & Kovacic (1972 ▶). For ring puckering analysis, see: Cremer & Pople (1975 ▶); Nardelli (1983 ▶).
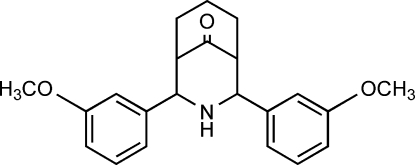


## Experimental

### 

#### Crystal data

C_22_H_25_NO_3_
*M* *_r_* = 351.43
Monoclinic, 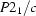

*a* = 22.3843 (9) Å
*b* = 6.5666 (3) Å
*c* = 13.0745 (4) Å
β = 106.382 (2)°
*V* = 1843.78 (13) Å^3^
*Z* = 4
Mo *K*α radiation
μ = 0.08 mm^−1^
*T* = 298 K
0.40 × 0.28 × 0.15 mm

#### Data collection

Bruker APEXII CCD area-detector diffractometer
Absorption correction: multi-scan (*SADABS*; Bruker, 1999 ▶) *T*_min_ = 0.967, *T*_max_ = 0.988
12835 measured reflections
3971 independent reflections
2326 reflections with *I* > 2σ(*I*)
*R*_int_ = 0.037

#### Refinement

*R*[*F* ^2^ > 2σ(*F* ^2^)] = 0.049
*wR*(*F* ^2^) = 0.136
*S* = 1.06
3971 reflections
241 parameters
H atoms treated by a mixture of independent and constrained refinement
Δρ_max_ = 0.30 e Å^−3^
Δρ_min_ = −0.23 e Å^−3^

### 

Data collection: *APEX2* (Bruker, 2004 ▶); cell refinement: *APEX2* and *SAINT-Plus* (Bruker, 2004 ▶); data reduction: *SAINT-Plus* and *XPREP* (Bruker, 2004 ▶); program(s) used to solve structure: *SHELXS97* (Sheldrick, 2008 ▶); program(s) used to refine structure: *SHELXL97* (Sheldrick, 2008 ▶); molecular graphics: *ORTEP-3* (Farrugia, 1997 ▶); software used to prepare material for publication: *SHELXL97*.

## Supplementary Material

Crystal structure: contains datablocks global, I. DOI: 10.1107/S1600536809050697/zl2238sup1.cif

Structure factors: contains datablocks I. DOI: 10.1107/S1600536809050697/zl2238Isup2.hkl

Additional supplementary materials:  crystallographic information; 3D view; checkCIF report

## Figures and Tables

**Table 1:** Hydrogen-bond geometry (Å, °)

*D*—H⋯*A*	*D*—H	H⋯*A*	*D*⋯*A*	*D*—H⋯*A*
N1—H1*N*⋯O1^i^	0.890 (18)	2.352 (18)	3.1901 (19)	157.0 (16)

Symmetry code: (i) 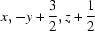.

**Fig. 1.**
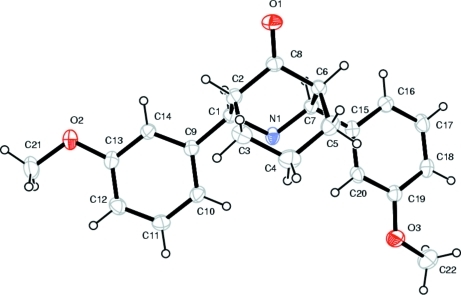
Anistropic displacement representation of the molecule with atoms represented with 30% probability ellipsoids.

**Fig. 2.**
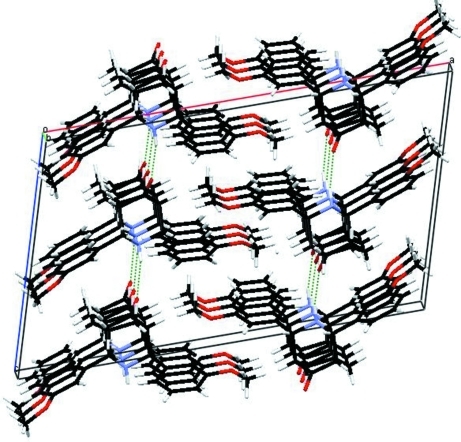
Packing diagram showing the N-H···O hydrogen bonding (green dashed lines) parallel to the b-axis.

## supplementary crystallographic information

### Comment

3-Azabicyclo[3.3.1]nonanes are an important class of heterocyclic compounds due to their broad-spectrum of biological activities such as analgesic, antogonistic, anti-inflammatory, local anesthetic and hypotensive activity (Jeyaraman & Avila, 1981). The 3-azabicyclo[3.3.1]nonane pharmacophore is present in numerous naturally occuring diterpenoid/norditerpenoid alkaloids such as methyllycaconitine, elatine, nudicauline, delsoline, delcorine and so on, they act as potential nicotinic acetylcholine receptor antagonists (Hardick *et al.* 1996; Barker *et al.* 2005). However, the biological activity mainly depends on the stereochemistry of the molecule; hence, it is of immense help to establish the structures of the synthesized molecules. For the synthesized title compound, several stereoisomers  are possible with conformations such as chair-chair (Parthiban *et al.*, 2008a, 2008b, 2008c, 2009a, 2009b, 2009c), chair-boat (Smith-Verdier *et al.*, 1983) and boat-boat (Padegimas & Kovacic, 1972). Hence, the present crystal study was undertaken to explore the configuration and conformation of the synthesized title compound.

The crystallographic analysis of the title compound shows that the piperidine ring adopts a near ideal chair conformation. The total puckering amplitude, Q_T_, is 0.600 (2) Å and the phase angle, θ, is 174.96 (19)° (Cremer & Pople, 1975). The smallest displacement asymmetry parameters being q2 and q3 are 0.053 (2) and -0.598 (2) Å (Nardelli, 1983). The deviation of ring atoms C8 and N1 from the C1/C2/C6/C7 plane are 0.712 (3) and -0.629 (3) Å, respectively.

According to the crystallographic analysis, the cyclohexane ring slightly deviates from the ideal chair conformation. The total puckering amplitude, Q_T_ = 0.559 (2) Å and phase angle θ = 166.6 (2)° (Cremer & Pople, 1975). The smallest displacement asymmetry parameters being q2 = 0.130 (2) and q3 = -0.544 (2)Å (Nardelli, 1983). The deviation of ring atoms C4 and C8 from the C2/C3/C5/C6 plane are -0.537 (4) and 0.718 (3) Å, respectively.

Hence the title compound, C_22_H_25_NO_3_, exists in a chair-chair conformation with equatorial orientation of the *meta*-methoxyphenyl groups on both sides of the secondary amino group on the heterocycle. The title compound is a positional isomer of 2,4-bis(2-methoxyphenyl)-3- azabicyclo[3.3.1]nonan-9-one (Parthiban *et al.*, 2009a) and 2,4-bis (4-methoxyphenyl)-3-azabicyclo[3.3.1]nonan-9-one (Cox *et al.*, 1985). Similar to the title compound the ortho as well as the para isomers also exhibit twin-chair conformations with equatorial disposition of the anisyl rings on both sides of the secondary amino group. In the title compound, the *meta*-methoxyphenyl rings are orientated at an angle of 25.02 (3)° with respect to one another whereas in the *ortho* and * para* isomer, the phenyl rings are orientated at an angle of 33.86 (3)° and 37.43 (4)° respectively.

The torsion angles of C8-C2-C1-C9 and C8-C6-C7-C15 are 179.64 (4) and 178.66 (3)°, respectively, for the title compound, which is very similar to those of its *ortho* isomer (-179.66 (3) and -179.76 (4)°, respectively) and those for the *para* isomer (178.2 (2) and 177.9 (4)°, respectively).

The crystal packing is dominated by shape recognition, by van der Waals interactions and is stabilized by an intermolecular N-H···O hydrogen bond (Table 1). In the *ortho* isomer, on the other hand, the crystal structure exhibits an intermolecular N-H···π interaction (N1-H1A···Cg1 = 2.75 Å).

### Experimental

The title compound was synthesized by a modified Mannich reaction using 0.1 mol (13.61 g/12.18 ml) *meta*-methoxybenzaldehyde, 0.05 mol (4.90 g/5.18 ml) cyclohexanone and 0.075 mol (5.78 g) ammonium acetate in 50 ml of absolute ethanol. The mixture was gently warmed on a hot plate with medium stirring and stirring was continued for about 15 h at a temperature of 303–308 K (30–35° C). After 12 h, the product formed was a spongy solid which was stirred for an additional 3 h until the reaction was complete as confirmed by the absence of aldehyde and cyclohexanone in the reaction mixture by TLC. After this, the crude compound was separated by filtration and washed with a 1:5 ethanol-ether mixture. X-ray diffraction quality crystals of 2,4-bis(3-methoxyphenyl)-3-azabicyclo[3.3.1]nonan-9-one were obtained by slow evaporation from ethanol.

### Refinement

The nitrogen H atom was located in a difference Fourier map and refined isotropically. Other hydrogen atoms were fixed geometrically and allowed to ride on the parent carbon atoms with aromatic C-H = 0.93 Å, aliphatic C-H = 0.98 Å, methylene C-H = 0.97 Å and methyl C-H = 0.96 Å. The displacement parameters were set for phenyl, methylene and aliphatic H atoms at *U*_iso_(H) = 1.2*U*_eq_(C) and for methyl H atoms at *U*_iso_(H) = 1.5*U*_eq_(C)

### Crystal data

**Table:**

C_22_H_25_NO_3_	*F*(000) = 752
*M**_r_* = 351.43	*D*_x_ = 1.266 Mg m^−^^3^
Monoclinic, *P*2_1_/*c*	Mo *K*α radiation, λ = 0.71073 Å
Hall symbol: -P 2ybc	Cell parameters from 2453 reflections
*a* = 22.3843 (9) Å	θ = 2.9–22.7°
*b* = 6.5666 (3) Å	µ = 0.08 mm^−^^1^
*c* = 13.0745 (4) Å	*T* = 298 K
β = 106.382 (2)°	Block, colourless
*V* = 1843.78 (13) Å^3^	0.40 × 0.28 × 0.15 mm
*Z* = 4	

### Data collection

**Table:**

Bruker APEXII CCD area-detector diffractometer	3971 independent reflections
Radiation source: fine-focus sealed tube	2326 reflections with *I* > 2σ(*I*)
graphite	*R*_int_ = 0.037
phi and ω scans	θ_max_ = 28.3°, θ_min_ = 2.9°
Absorption correction: multi-scan (*SADABS*; Bruker, 1999)	*h* = −27→28
*T*_min_ = 0.967, *T*_max_ = 0.988	*k* = −7→8
12835 measured reflections	*l* = −12→17

### Refinement

**Table:**

Refinement on *F*^2^	Primary atom site location: structure-invariant direct methods
Least-squares matrix: full	Secondary atom site location: difference Fourier map
*R*[*F*^2^ > 2σ(*F*^2^)] = 0.049	Hydrogen site location: inferred from neighbouring sites
*wR*(*F*^2^) = 0.136	H atoms treated by a mixture of independent and constrained refinement
*S* = 1.06	*w* = 1/[σ^2^(*F*_o_^2^) + (0.0643*P*)^2^] where *P* = (*F*_o_^2^ + 2*F*_c_^2^)/3
3971 reflections	(Δ/σ)_max_ < 0.001
241 parameters	Δρ_max_ = 0.30 e Å^−^^3^
0 restraints	Δρ_min_ = −0.23 e Å^−^^3^

### Special details

**Table:**

Geometry. All esds (except the esd in the dihedral angle between two l.s. planes) are estimated using the full covariance matrix. The cell esds are taken into account individually in the estimation of esds in distances, angles and torsion angles; correlations between esds in cell parameters are only used when they are defined by crystal symmetry. An approximate (isotropic) treatment of cell esds is used for estimating esds involving l.s. planes.
Refinement. Refinement of F^2^ against ALL reflections. The weighted R-factor wR and goodness of fit S are based on F^2^, conventional R-factors R are based on F, with F set to zero for negative F^2^. The threshold expression of F^2^ > 2sigma(F^2^) is used only for calculating R-factors(gt) etc. and is not relevant to the choice of reflections for refinement. R-factors based on F^2^ are statistically about twice as large as those based on F, and R- factors based on ALL data will be even larger.

### Fractional atomic coordinates and isotropic or equivalent isotropic displacement parameters (Å^2^)

**Table:**

	*x*	*y*	*z*	*U*_iso_*/*U*_eq_	
C1	0.32467 (7)	0.5186 (3)	0.49436 (13)	0.0356 (4)	
H1	0.3471	0.6483	0.5098	0.043*	
C2	0.30802 (8)	0.4800 (3)	0.37207 (13)	0.0388 (5)	
H2	0.3464	0.4857	0.3502	0.047*	
C3	0.27531 (9)	0.2775 (3)	0.33443 (15)	0.0479 (5)	
H3A	0.2724	0.2589	0.2596	0.057*	
H3B	0.3005	0.1674	0.3737	0.057*	
C4	0.21054 (10)	0.2635 (3)	0.34870 (16)	0.0573 (6)	
H4A	0.1892	0.1478	0.3085	0.069*	
H4B	0.2141	0.2393	0.4234	0.069*	
C5	0.17173 (9)	0.4527 (3)	0.31292 (15)	0.0484 (5)	
H5A	0.1356	0.4469	0.3398	0.058*	
H5B	0.1569	0.4529	0.2357	0.058*	
C6	0.20648 (8)	0.6525 (3)	0.34984 (13)	0.0398 (4)	
H6	0.1807	0.7660	0.3133	0.048*	
C7	0.22453 (7)	0.6922 (3)	0.47199 (13)	0.0376 (4)	
H7	0.2460	0.8237	0.4860	0.045*	
C8	0.26600 (8)	0.6491 (3)	0.31776 (13)	0.0382 (4)	
C9	0.36693 (7)	0.3516 (3)	0.55456 (12)	0.0351 (4)	
C10	0.34445 (8)	0.1866 (3)	0.59844 (13)	0.0430 (5)	
H10	0.3024	0.1796	0.5947	0.052*	
C11	0.38393 (9)	0.0330 (3)	0.64743 (15)	0.0495 (5)	
H11	0.3682	−0.0761	0.6771	0.059*	
C12	0.44674 (9)	0.0379 (3)	0.65342 (14)	0.0489 (5)	
H12	0.4732	−0.0664	0.6869	0.059*	
C13	0.46919 (8)	0.2005 (3)	0.60884 (14)	0.0419 (5)	
C14	0.42955 (8)	0.3570 (3)	0.56051 (13)	0.0386 (4)	
H14	0.4453	0.4671	0.5317	0.046*	
C15	0.16734 (8)	0.7034 (3)	0.51186 (13)	0.0371 (4)	
C16	0.13403 (8)	0.8838 (3)	0.50128 (15)	0.0473 (5)	
H16	0.1471	0.9958	0.4697	0.057*	
C17	0.08192 (9)	0.8993 (3)	0.53688 (16)	0.0532 (5)	
H17	0.0604	1.0220	0.5296	0.064*	
C18	0.06116 (8)	0.7346 (3)	0.58332 (15)	0.0478 (5)	
H18	0.0258	0.7451	0.6070	0.057*	
C19	0.09401 (8)	0.5548 (3)	0.59381 (14)	0.0409 (5)	
C20	0.14652 (8)	0.5399 (3)	0.55826 (14)	0.0403 (5)	
H20	0.1681	0.4173	0.5658	0.048*	
C21	0.57145 (9)	0.0580 (3)	0.65086 (19)	0.0692 (7)	
H21A	0.5747	0.0431	0.7253	0.104*	
H21B	0.6118	0.0868	0.6423	0.104*	
H21C	0.5557	−0.0659	0.6141	0.104*	
C22	0.03130 (10)	0.3935 (4)	0.69181 (18)	0.0737 (7)	
H22A	0.0429	0.4937	0.7474	0.111*	
H22B	0.0268	0.2633	0.7223	0.111*	
H22C	−0.0075	0.4317	0.6421	0.111*	
N1	0.26760 (6)	0.5346 (2)	0.52819 (12)	0.0370 (4)	
O1	0.27803 (6)	0.7680 (2)	0.25553 (10)	0.0548 (4)	
O2	0.53008 (6)	0.2212 (2)	0.60764 (11)	0.0612 (4)	
O3	0.07815 (6)	0.3815 (2)	0.63810 (11)	0.0613 (4)	
H1N	0.2775 (8)	0.558 (3)	0.5980 (15)	0.048 (6)*	

### Atomic displacement parameters (Å^2^)

**Table:**

	*U*^11^	*U*^22^	*U*^33^	*U*^12^	*U*^13^	*U*^23^
C1	0.0318 (9)	0.0366 (11)	0.0398 (10)	0.0004 (8)	0.0123 (8)	−0.0011 (8)
C2	0.0399 (10)	0.0469 (12)	0.0347 (10)	0.0054 (9)	0.0190 (8)	0.0037 (8)
C3	0.0630 (13)	0.0441 (12)	0.0381 (10)	0.0039 (10)	0.0168 (9)	−0.0040 (9)
C4	0.0650 (14)	0.0463 (13)	0.0560 (13)	−0.0119 (11)	0.0097 (11)	−0.0059 (10)
C5	0.0440 (11)	0.0584 (14)	0.0405 (11)	−0.0084 (10)	0.0083 (9)	−0.0056 (10)
C6	0.0372 (10)	0.0426 (11)	0.0390 (10)	0.0050 (8)	0.0096 (8)	0.0064 (9)
C7	0.0341 (9)	0.0372 (11)	0.0428 (10)	0.0001 (8)	0.0128 (8)	−0.0016 (8)
C8	0.0431 (10)	0.0402 (11)	0.0314 (9)	−0.0028 (9)	0.0107 (8)	0.0010 (9)
C9	0.0369 (10)	0.0408 (11)	0.0293 (9)	0.0022 (8)	0.0120 (7)	−0.0016 (8)
C10	0.0375 (10)	0.0547 (13)	0.0401 (10)	0.0012 (9)	0.0165 (8)	0.0066 (9)
C11	0.0554 (12)	0.0529 (13)	0.0456 (12)	0.0049 (10)	0.0229 (10)	0.0154 (10)
C12	0.0512 (12)	0.0535 (13)	0.0420 (11)	0.0125 (10)	0.0133 (9)	0.0123 (10)
C13	0.0333 (10)	0.0524 (13)	0.0400 (10)	0.0034 (9)	0.0101 (8)	−0.0009 (9)
C14	0.0362 (10)	0.0417 (11)	0.0392 (10)	0.0000 (8)	0.0128 (8)	0.0021 (9)
C15	0.0339 (9)	0.0407 (11)	0.0357 (9)	0.0034 (8)	0.0081 (8)	−0.0037 (8)
C16	0.0430 (11)	0.0400 (12)	0.0612 (13)	0.0041 (9)	0.0184 (9)	0.0037 (10)
C17	0.0451 (12)	0.0464 (13)	0.0692 (14)	0.0152 (10)	0.0177 (10)	0.0001 (11)
C18	0.0355 (10)	0.0562 (14)	0.0537 (12)	0.0086 (10)	0.0162 (9)	−0.0021 (10)
C19	0.0383 (10)	0.0435 (12)	0.0414 (11)	0.0041 (9)	0.0121 (8)	0.0005 (9)
C20	0.0382 (10)	0.0389 (11)	0.0452 (11)	0.0104 (8)	0.0140 (8)	0.0008 (9)
C21	0.0418 (12)	0.0740 (17)	0.0868 (17)	0.0182 (11)	0.0098 (11)	0.0064 (13)
C22	0.0694 (15)	0.0840 (19)	0.0840 (17)	0.0043 (13)	0.0481 (14)	0.0144 (14)
N1	0.0325 (8)	0.0488 (10)	0.0313 (9)	0.0070 (7)	0.0114 (7)	−0.0019 (7)
O1	0.0582 (9)	0.0581 (10)	0.0507 (8)	−0.0021 (7)	0.0194 (7)	0.0200 (7)
O2	0.0350 (8)	0.0656 (10)	0.0832 (11)	0.0114 (7)	0.0167 (7)	0.0151 (8)
O3	0.0600 (9)	0.0554 (10)	0.0812 (10)	0.0095 (7)	0.0406 (8)	0.0162 (8)

### Geometric parameters (Å, °)

**Table:**

C1—N1	1.469 (2)	C11—C12	1.386 (3)
C1—C9	1.516 (2)	C11—H11	0.9300
C1—C2	1.557 (2)	C12—C13	1.378 (3)
C1—H1	0.9800	C12—H12	0.9300
C2—C8	1.498 (2)	C13—O2	1.374 (2)
C2—C3	1.531 (2)	C13—C14	1.388 (2)
C2—H2	0.9800	C14—H14	0.9300
C3—C4	1.516 (3)	C15—C20	1.378 (2)
C3—H3A	0.9700	C15—C16	1.385 (2)
C3—H3B	0.9700	C16—C17	1.376 (2)
C4—C5	1.512 (3)	C16—H16	0.9300
C4—H4A	0.9700	C17—C18	1.383 (3)
C4—H4B	0.9700	C17—H17	0.9300
C5—C6	1.532 (2)	C18—C19	1.378 (2)
C5—H5A	0.9700	C18—H18	0.9300
C5—H5B	0.9700	C19—O3	1.368 (2)
C6—C8	1.506 (2)	C19—C20	1.384 (2)
C6—C7	1.555 (2)	C20—H20	0.9300
C6—H6	0.9800	C21—O2	1.425 (2)
C7—N1	1.463 (2)	C21—H21A	0.9600
C7—C15	1.514 (2)	C21—H21B	0.9600
C7—H7	0.9800	C21—H21C	0.9600
C8—O1	1.2119 (19)	C22—O3	1.419 (2)
C9—C14	1.382 (2)	C22—H22A	0.9600
C9—C10	1.386 (2)	C22—H22B	0.9600
C10—C11	1.374 (2)	C22—H22C	0.9600
C10—H10	0.9300	N1—H1N	0.890 (18)
			
N1—C1—C9	111.34 (13)	C11—C10—H10	119.8
N1—C1—C2	110.15 (13)	C9—C10—H10	119.8
C9—C1—C2	110.43 (13)	C10—C11—C12	121.21 (18)
N1—C1—H1	108.3	C10—C11—H11	119.4
C9—C1—H1	108.3	C12—C11—H11	119.4
C2—C1—H1	108.3	C13—C12—C11	118.63 (18)
C8—C2—C3	108.17 (15)	C13—C12—H12	120.7
C8—C2—C1	107.60 (13)	C11—C12—H12	120.7
C3—C2—C1	115.21 (14)	O2—C13—C12	124.24 (17)
C8—C2—H2	108.6	O2—C13—C14	115.51 (16)
C3—C2—H2	108.6	C12—C13—C14	120.25 (16)
C1—C2—H2	108.6	C9—C14—C13	120.99 (17)
C4—C3—C2	113.59 (15)	C9—C14—H14	119.5
C4—C3—H3A	108.8	C13—C14—H14	119.5
C2—C3—H3A	108.8	C20—C15—C16	118.10 (16)
C4—C3—H3B	108.8	C20—C15—C7	122.51 (16)
C2—C3—H3B	108.8	C16—C15—C7	119.40 (16)
H3A—C3—H3B	107.7	C17—C16—C15	120.87 (18)
C5—C4—C3	113.46 (16)	C17—C16—H16	119.6
C5—C4—H4A	108.9	C15—C16—H16	119.6
C3—C4—H4A	108.9	C16—C17—C18	120.83 (18)
C5—C4—H4B	108.9	C16—C17—H17	119.6
C3—C4—H4B	108.9	C18—C17—H17	119.6
H4A—C4—H4B	107.7	C19—C18—C17	118.55 (17)
C4—C5—C6	114.18 (15)	C19—C18—H18	120.7
C4—C5—H5A	108.7	C17—C18—H18	120.7
C6—C5—H5A	108.7	O3—C19—C18	124.06 (16)
C4—C5—H5B	108.7	O3—C19—C20	115.45 (16)
C6—C5—H5B	108.7	C18—C19—C20	120.48 (17)
H5A—C5—H5B	107.6	C15—C20—C19	121.17 (16)
C8—C6—C5	108.04 (15)	C15—C20—H20	119.4
C8—C6—C7	107.14 (13)	C19—C20—H20	119.4
C5—C6—C7	115.39 (14)	O2—C21—H21A	109.5
C8—C6—H6	108.7	O2—C21—H21B	109.5
C5—C6—H6	108.7	H21A—C21—H21B	109.5
C7—C6—H6	108.7	O2—C21—H21C	109.5
N1—C7—C15	111.28 (14)	H21A—C21—H21C	109.5
N1—C7—C6	109.93 (14)	H21B—C21—H21C	109.5
C15—C7—C6	111.21 (13)	O3—C22—H22A	109.5
N1—C7—H7	108.1	O3—C22—H22B	109.5
C15—C7—H7	108.1	H22A—C22—H22B	109.5
C6—C7—H7	108.1	O3—C22—H22C	109.5
O1—C8—C2	124.55 (16)	H22A—C22—H22C	109.5
O1—C8—C6	123.99 (16)	H22B—C22—H22C	109.5
C2—C8—C6	111.46 (14)	C7—N1—C1	113.91 (13)
C14—C9—C10	118.53 (17)	C7—N1—H1N	109.3 (12)
C14—C9—C1	119.04 (16)	C1—N1—H1N	109.6 (11)
C10—C9—C1	122.31 (15)	C13—O2—C21	117.20 (16)
C11—C10—C9	120.39 (17)	C19—O3—C22	118.64 (15)
			
N1—C1—C2—C8	56.22 (18)	C10—C11—C12—C13	−0.1 (3)
C9—C1—C2—C8	179.60 (13)	C11—C12—C13—O2	−178.66 (17)
N1—C1—C2—C3	−64.51 (19)	C11—C12—C13—C14	1.0 (3)
C9—C1—C2—C3	58.88 (19)	C10—C9—C14—C13	0.3 (3)
C8—C2—C3—C4	−53.64 (19)	C1—C9—C14—C13	−175.81 (15)
C1—C2—C3—C4	66.8 (2)	O2—C13—C14—C9	178.61 (16)
C2—C3—C4—C5	45.2 (2)	C12—C13—C14—C9	−1.0 (3)
C3—C4—C5—C6	−44.8 (2)	N1—C7—C15—C20	−25.2 (2)
C4—C5—C6—C8	52.4 (2)	C6—C7—C15—C20	97.7 (2)
C4—C5—C6—C7	−67.4 (2)	N1—C7—C15—C16	155.10 (16)
C8—C6—C7—N1	−57.64 (18)	C6—C7—C15—C16	−82.0 (2)
C5—C6—C7—N1	62.69 (18)	C20—C15—C16—C17	0.5 (3)
C8—C6—C7—C15	178.66 (15)	C7—C15—C16—C17	−179.85 (16)
C5—C6—C7—C15	−61.0 (2)	C15—C16—C17—C18	−0.6 (3)
C3—C2—C8—O1	−116.08 (19)	C16—C17—C18—C19	0.4 (3)
C1—C2—C8—O1	118.86 (18)	C17—C18—C19—O3	−179.87 (18)
C3—C2—C8—C6	63.68 (17)	C17—C18—C19—C20	−0.2 (3)
C1—C2—C8—C6	−61.37 (18)	C16—C15—C20—C19	−0.3 (3)
C5—C6—C8—O1	116.90 (19)	C7—C15—C20—C19	−179.91 (15)
C7—C6—C8—O1	−118.19 (18)	O3—C19—C20—C15	179.84 (16)
C5—C6—C8—C2	−62.87 (18)	C18—C19—C20—C15	0.1 (3)
C7—C6—C8—C2	62.04 (19)	C15—C7—N1—C1	−178.96 (13)
N1—C1—C9—C14	−158.74 (15)	C6—C7—N1—C1	57.38 (18)
C2—C1—C9—C14	78.57 (19)	C9—C1—N1—C7	−179.40 (14)
N1—C1—C9—C10	25.3 (2)	C2—C1—N1—C7	−56.55 (19)
C2—C1—C9—C10	−97.36 (18)	C12—C13—O2—C21	3.2 (3)
C14—C9—C10—C11	0.5 (3)	C14—C13—O2—C21	−176.39 (17)
C1—C9—C10—C11	176.49 (16)	C18—C19—O3—C22	−10.4 (3)
C9—C10—C11—C12	−0.6 (3)	C20—C19—O3—C22	169.87 (17)

### Hydrogen-bond geometry (Å, °)

**Table:**

*D*—H···*A*	*D*—H	H···*A*	*D*···*A*	*D*—H···*A*
N1—H1N···O1^i^	0.890 (18)	2.352 (18)	3.1901 (19)	157.0 (16)

Symmetry codes: (i) *x*, −*y*+3/2, *z*+1/2.
